# Health indicators and human development in the Arab region

**DOI:** 10.1186/1476-072X-5-61

**Published:** 2006-12-28

**Authors:** Abdesslam Boutayeb, Mansour Serghini

**Affiliations:** 1 Department of Mathematics, University Mohammed Ier, BP: 717, Oujda, Morocco

## Abstract

**Background:**

The present paper deals with the relationship between health indicators and human development in the Arab region. Beyond descriptive analysis showing geographic similarities and disparities inter countries, the main purpose is to point out health deficiencies and to propose pragmatic strategies susceptible to improve health conditions and consequently enhance human development in the Arab world.

**Methods:**

Data analysis using Principal Components Analysis is used to compare the achievements of the Arab countries in terms of direct and indirect health indicators. The variables (indicators) are seen to be well represented on the circle of correlation, allowing for interesting interpretation and analysis. The 19 countries are projected on the first and second plane respectively.

**Results:**

The results given by the present analysis give a good panorama of the Arab countries with their geographic similarities and disparities. The high correlation between health indicators and human development is well illustrated and consequently, countries are classified by groups having similar human development. The analysis shows clearly how health deficits are impeding human development in the majority of Arab countries and allows us to formulate suggestions to improve health conditions and enhance human development in the Arab World.

**Discussion:**

The discussion is based on the link between different direct and indirect health indicators and the relationship between these indicators and human development index. Without including the GDP indicator, our analysis has shown that the 19 Arab countries may be classified, independently of their geographic proximity, in three different groups according to their global human development level (Low, Medium and High). Consequently, while identifying health deficiencies in each group, the focus was made on the countries presenting a high potential of improvement in health indicators. In particular, maternal mortality and infant mortality which are really challenging health authorities of the first and third group were critically discussed.

**Conclusion:**

The Arab countries have made substantial economic and social progress during the last decades by improving life expectancy and reducing maternal and infant mortality. However, considering its natural wealth and human resources, the Arab region has accomplished less than expected in terms of human development. Huge social inequalities and health inequities exist inter and intra Arab countries. In most Arab countries, a large percentage of populations, especially in rural areas, are deprived of access to health facilities. Consequently, many women still die during pregnancy and labour, yielding unacceptable levels of maternal and infant mortality. However, the problem is seen to be more complex, going beyond geography and technical accessibility to health care, it compasses, among others, levels of literacy, low social and economic status of women, qualification of health staff, general behaviour and interactions between patients and medical personnel (including corruption).

## Background

In 2000, the population of the 22 Arab countries was about 280 million representing approximately 5% of the world population. In a series of reports, The United Nations Development Program (UNDP) considered the human development globally in the Arab world. The first Arab Human Development Report (AHDR 2002) [1] diagnosed three cardinal deficits impeding human development in Arab countries (knowledge acquisition, freedom and good governance, and woman's empowerment). Accordingly, the second report (AHDR 2003) [2] was devoted to the knowledge acquisition deficit whereas the third report (AHDR 2004) [3] dealt with the deficit in freedom and good governance. Finally, the question of woman's empowerment was examined by the fourth and last report in the series (AHDR 2005) [4]. While the importance of analysing in depth these three deficits is indisputable, and besides the interesting and controversial debate engaged among Arab intellectuals [5-9] by these reports as political platforms rather than academic or scientific ones, it should be stressed that the question of health has been somehow relegated to a second order of priority.

In developing countries in general and in Arab countries in particular, until recently, it was widely believed that economic development was a necessary prerequisite for improving a population health status and health was often classified as a non productive sector. But recent evidence showed that health is more than a consequence of development, it is a central input into social and economic development and poverty reduction [10-14].

Measurable indicators are playing useful roles in identifying problems, determining trends and suggesting pragmatic strategies [15]. In particular, human development index (HDI) is the most used index giving a summary measure of human development and allowing for comparison between countries around the world. HDI is a three dimensional composite index obtained as a mean of three indicators weighted equally: health (life expectancy at birth), standard of living (GDP per capita) and education (literacy and enrolment) [16]. In this paper, considering health as part of human development, the main objective is the study of direct and indirect health indicators in the 22 countries constituting the Arab world in order to see how these indicators are affecting the Arab human development globally and geographically country by country. Quantitative and qualitative comparison will be made among Arab countries according to data (Table 1) on life expectancy at birth (LEB), infant mortality (IMR), maternal mortality (MMR), expectation of lost healthy years (ELHY), deliveries attended by skilled attendants (DASA), pregnant women receiving prenatal care (PWRP), number of physicians (PPP), percentage of children under weight (CUW) and data related indirectly to health such as percentages of literacy in male and female (Lm, Lf) and percentages of enrolment (Enm, Enf). Taking into account the fact that Arab countries share geographic proximity, language, culture and tradition, the following questions will be addressed: 1 – What are the most significant indicators illustrating geographic similarities and differences? 2 – How are different indicators related? 3 – Do Arab countries constitute a homogeneous group? 4 – How are social inequalities, health inequities and geographic disparities affecting the health status of the Arab populations? 5 – What suggestions can globally be made to help health decision makers in order to improve health conditions by reducing the burden of disease, disability and mortality in the Arab region?

**Table 1:** Health indicators

		**Life expectancy at birth **(years) 2002		**expectation of lost healthy 2002**		**Maternal Mortality Ratio per 100000–2000**	**delivery attended by skilled attendant 1996 (%)**	**Preghant women who received prenatal care 1996 (%)**	**Children under weitht % of <5 y old 1995–2002**	**Infant mortality rates per 1000 live births 2002**	**Physicians Per 100000 people 1990–2003**	**HDI rank 2002 Rank/177 countries**	**Literacy (%) 2002**		**Enrolment (%) 2001/2002**	
		Female	Male	Female	Male								Male	femal	Male	female

		LEBf	LEBm	ELHf	ELHm	MMR	DASA	PWRP	CUW	IMR	PPP	HDI	Lm	Lf	Enm	Enf

Algeria	Alg	71,1	68	9,6	7,9	140	77	58	5	39	85	108	78	59,6	72	69
Bahrrain	Bah	75,8	72,4	10,1	7,9	28	94	96	9	13	169	40	91,5	84,2	77	82
Comoros	Com	62	59,2	9,6	7,8	480	24	69	25	59	7	136	63,5	49,1	50	41
Djibouti	Dji	47	44,8	7,4	6,1	730	79	76	18	100	13	154	76,1	55,5	28	20
Egypt	Egy	70,8	66,6	8,8	7,4	84	46	53	11	35	218	120	67,2	43,6	80	72
Irak	Ira			11,6	10,3	250	54	59		102						
Jordan	Jor	72,4	69,6	10,9	9	41	87	80	5	27	205	90	95,5	85,9	76	77
Kuwait	Kuw	78,9	74,8	10,6	8,2	5	99	99	10	9	160	44	84,7	81	71	81
Lebanon	Leb	75	71,8	10,4	8,4	150	45	85	3	28	274	80	92,4	81	77	79
Libya	Lib	75,3	70,7	10,5	8,1	97	76	100	5	16	120	58	91,8	70,7	93	100
Mauritani	Mau	53,9	50,7	8,2	6,9	1000	40	49	32	120	14	152	51,5	31,3	46	42
Morocco	Mor	70,3	66,6	11,9	9,4	220	40	45	9	39	49	125	63,3	38,3	61	52
Palestine	Pal	73,9	70,7						4	23		102			78	81
Oman	Oma	74,3	70,9	11,1	8,3	87	92	98	24	11	137	74	82	65,4	62	63
Qatar	Qat	75,3	70,4	10	8,2	7	97	100	6	11	220	47	84,9	82,3	79	84
Saudi Arabia	Sau	73,6	71	11	8,6	23	90	87	14	23	153	77	84,1	69,5	58	57
Somalia	Som			8,1	6,9	1100	2	40		133						
Sudan	Sud	57	54,1	9,4	7,8	590	86	54	17	64	16	139	70,8	49,1	39	34
Syria	Syr	73	70,5	10,5	8,5	160	67	33	7	23	142	106	91	74,2	62	57
Tunisia	Tun	74,8	70,7	10,3	8,2	120	90	71	4	21	70	92	83,1	63,1	74	75
UAE	UAE	77,3	73,2	10,9	7,8	54	96	95	14	8	177	49	80,7	75,6	65	72
Yemen	Yem	60,9	58,7	11,5	10,8	570	16	26	46	79	22	149	69,5	28,5	66	37

Data with the abbreviated names of countries

## Methods

In order to get a summary of information on the interaction between all variables (indicators) at the same time, Principal Components Analysis (PCA) is used to select the first and second factors (F1 and F2). The corresponding eigenvalues give the percentages of information summarised by these factors respectively. Variables are then projected on the circle of correlation and countries on the first plane (F1 × F2). The more the variables are near the circle, the better will be the interpretation. In the meantime, a projection of countries on the first plane should lead to a classification according to the scores of each country with regard to the variables under consideration. Additional information may be obtained by the third and fourth axis, but the interpretation is then conditional to what was given by the precedent factors.

Data are missing for some countries (Irak, Palestine & Somalia). Consequently, PCA is applied with 19 countries only.

Table 1 shows health indicators considered as variables in PCA. Since one of our objectives is to study how these variables are affecting human development in the Arab world, the variable Human Development Index (HDI) is not included in the analysis but it is projected as a supplementary variable. Considering that literacy and enrolment are important indictors acting indirectly on and with health indicators, the variables (Lm, Lf, Enm and Enm) are also included in the analysis.

## Results

**Figure 1:**
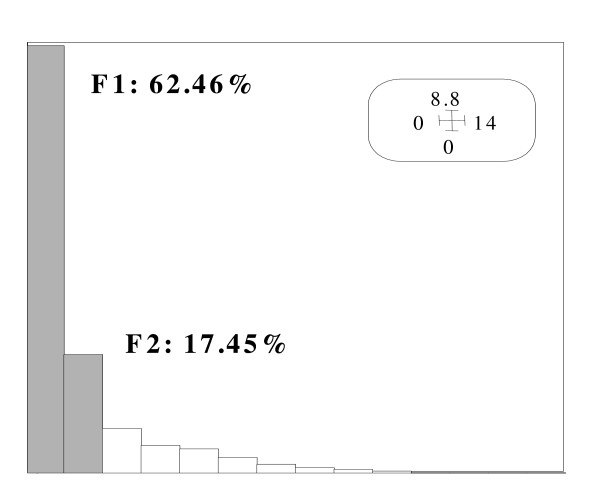
**Histogram of the eigenvalues**. Percentage of information explained.

As indicated in Figure 1, the contribution of the first (F1) and the second (F2) principal axis are respectively 62.46% and 17.45% yielding a first plane (F1 × F2) that summarizes information to an extent of 80%.

**Figure 2:**
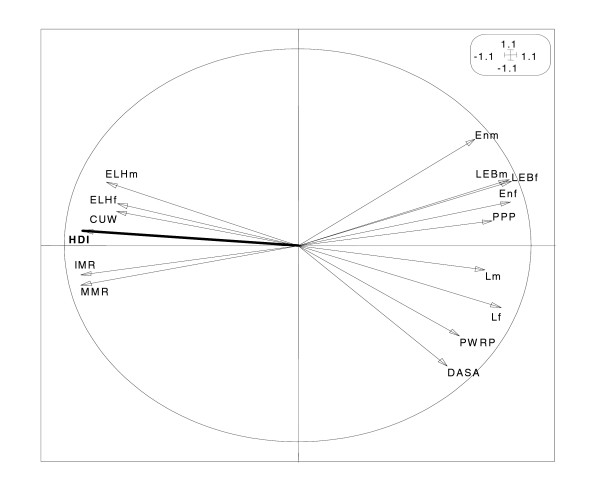
**Correlations on the first plane**. Correlation circle of indicators on the first factorial plane (F1 × F2) of the principal components analysis.

All the variables are well represented on the circle of correlations shown in Figure 2, allowing for interesting interpretation. The variables IMR, MMR, CUV, ELHf and ELHm are positively correlated each to other, with the correlation between IMR and MMR reaching .97. These five variables are negatively correlated to the other variables (DASA, PWRP, Lf, Lm, PPP, Enf, LEBf, LEBm and Enm). The graphic illustration is confirmed by the numbers given in the correlation matrix (Table 2).

**Table 2:** Correlation matrix

LEBf	1.0													
LEBm	.99	1.0												
ELHf	-.59	-.56	1.0											
ELHm	-.65	-.63	.93	1.0										
MMR	-.94	-.94	.59	.66	1.0									
DASA	.43	.42	-.50	-.62	-.53	1.0								
PWRP	.49	.47	-.54	-.67	-.53	.68	1.0							
CUW	-.60	-.59	.70	.74	.68	-.54	-.42	1.0						
IMR	-.95	-.95	.57	.67	.97	-.57	-.58	.65	1.0					
PPP	.75	.74	-.66	-.63	-.76	.37	.52	-.59	-.72	1.0				
Lm	.65	.67	-.44	-.49	-.72	.62	.56	-.63	-.73	.67	1.0			
Lf	.68	.69	-.62	-.69	-.73	.72	.74	-.69	-.75	.74	.90	1.0		
Enm	.81	.80	-.54	-.44	-.75	.15	.33	-.49	-.71	.68	.55	.47	1.0	
Enf	.87	.85	-.72	-.70	-.81	.43	.60	-.67	-.81	.75	.65	.69	.92	1.0

Correlations between different indicators

**Figure 3:**
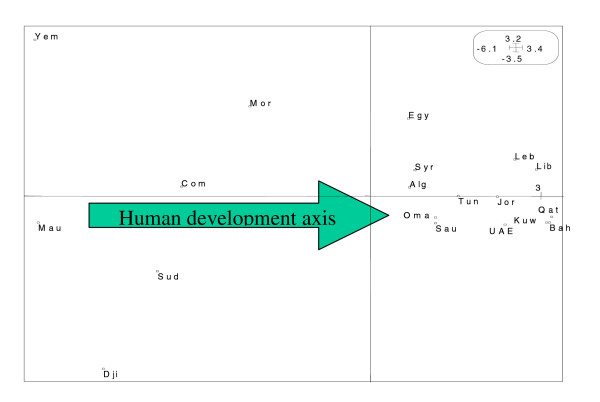
**Countries on the first plane**. Projection of countries on the first factorial plane (F1 × F2) of the principal components analysis.

Projection of the 19 countries on the first plane (F1 × F2) allows us to reconstitute the classification of Arab countries in term of their HDI. Figure 3 shows clearly the horizontal sketch of countries. Those having the least human development (LHD) Comoros (Com), Djibouti (Dji), Mauritania (Mau), Sudan (Sud) and Yemen (Yem) are opposed to those with the highest human development (HHD) in the Arab world Bahrain (Bah), Kuwait (Kuw), Lybia (Lyb), Qatar (Qat) and United Arab Emirates (UAE), while a third group containing Algeria (Alg), Egypt (Egy), Jordan (Jor), Lebanon (Leb), Morocco (Mor), Oman (Oma), Saudi Arabia (Sau), Syria (Syr) and Tunisia (Tun) constitutes an intermediate class (MHD). We have thus answered the first questions by showing the correlations exiting positively or negatively between different indicators and reproducing globally the large disparities between Arab countries, independently of geographic proximity.

Without using the GDP indicator, our result agrees fairly with the three classes of human development (high, medium and low) given by the United Nation Development Programme in the 2003 report [17].

Looking at the poor and least developed group (LHD) (Table 3), it is striking to see how the five countries seem similar in that they share low levels of life expectancy and high levels of maternal mortality, infant mortality, percentage of children under weight and number of lost healthy years. In fact these non-oil countries share also low income, low economic growth, low access to health care, drinking water and sanitation, and except Djibouti, they have high percentages of rural populations [18,19]. A special attention needs to be drawn to the case of Djibouti which has the lowest life expectancy and the second highest level of maternal and infant mortality. In this country, the prevalence of HIV/AIDS (2 to 5%) is exceptionally high comparatively to the group of Arab countries known to have one of the smallest prevalence of HIV/AIDS in the world [20,21].

**Table 3:** Group 1

		**Life expectancy at birth **(years) 2002		**expectation of lost healthy 2002**		**Maternal Mortality Ratio per 100000–2000**	**delivery attended by skilled attendant 1996 (%)**	**Preghant women who received prenatal care 1996 (%)**	**Children under weitht % of <5 y old 1995–2002**	**Infant mortality rates per 1000 live births 2002**	**Physicians Per 100000 people 1990–2003**	**HDI rank 2002 Rank/177 countries**	**Literacy (%) 2002**		**Enrolment (%) 2001/2002**	
		Female	Male	Female	Male								Male	femal	Male	female

		LEBf	LEBm	ELHf	ELHm	MMR	DASA	PWRP	CUW	IMR	PPP	HDI	Lm	Lf	Enm	Enf

Comoros	Com	62	59,2	9,6	7,8	480	24	69	25	59	7	136	63,5	49,1	50	41
Djibouti	Dji	47	44,8	7,4	6,1	730	79	76	18	100	13	154	76,1	55,5	28	20
Mauritani	Mau	53,9	50,7	8,2	6,9	1000	40	49	32	120	14	152	51,5	31,3	46	42
Sudan	Sud	57	54,1	9,4	7,8	590	86	54	17	64	16	139	70,8	49,1	39	34
Yemen	Yem	60,9	58,7	11,5	10,8	570	16	26	46	79	22	149	69,5	28,5	66	37

Low Human Development(LHD)

On the opposite side, with the best human development (HHD) (Table 4), we find a group of oil-rich countries formed by Bahrain, Kuwait, Libya, Qatar and United Arab Emirates. With small sized and highly urbanized populations, the five countries share high income and similar pattern of human development. A part from few exceptions, they have high life expectancy, low levels of: lost healthy years, maternal mortality, infant mortality and children under weight. Populations of these countries have good access to education and health.

**Table 4:** Group 2

		**Life expectancy at birth **(years) 2002		**expectation of lost healthy 2002**		**Maternal Mortality Ratio per 100000–2000**	**delivery attended by skilled attendant 1996 (%)**	**Preghant women who received prenatal care 1996 (%)**	**Children under weitht % of <5 y old 1995–2002**	**Infant mortality rates per 1000 live births 2002**	**Physicians Per 100000 people 1990–2003**	**HDI rank 2002 Rank/177 countries**	**Literacy (%) 2002**		**Enrolment (%) 2001/2002**	
		Female	Male	Female	Male								Male	femal	Male	female

		LEBf	LEBm	ELHf	ELHm	MMR	DASA	PWRP	CUW	IMR	PPP	HDI	Lm	Lf	Enm	Enf

Bahrrain	Bah	75,8	72,4	10,1	7,9	28	94	96	9	13	169	40	91,5	84,2	77	82
Kuwait	Kuw	78,9	74,8	10,6	8,2	5	99	99	10	9	160	44	84,7	81	71	81
Libya	Lib	75,3	70,7	10,5	8,1	97	76	100	5	16	120	58	91,8	70,7	93	100
Qatar	Qat	75,3	70,4	10	8,2	7	97	100	6	11	220	47	84,9	82,3	79	84
UAE	UAE	77,3	73,2	10,9	7,8	54	96	95	14	8	177	49	80,7	75,6	65	72

High Human Development (HHD)

Finally, the intermediate group (MHD) (Table 5) is rather heterogeneous with regards to many components. Although the nine countries fall all in the medium income class according to the World Bank classification [19], we find in the same group oil- and non oil-producers, large and small sizes of populations, high and low levels of maternal mortality, infant mortality, education and other indicators. In this group, the low-medium income and least developed are Morocco, Egypt, Algeria and Syria, opposed to the relatively best developed: Oman, Saudi Arabia, Lebanon, Jordan and Tunisia. Two third of the Arab population are living in these countries.

**Table 5:** Group 3

		**Life expectancy at birth **(years) 2002		**expectation of lost healthy 2002**		**Maternal Mortality Ratio per 100000–2000**	**delivery attended by skilled attendant 1996 (%)**	**Preghant women who received prenatal care 1996 (%)**	**Children under weitht % of <5 y old 1995–2002**	**Infant mortality rates per 1000 live births 2002**	**Physicians Per 100000 people 1990–2003**	**HDI rank 2002 Rank/177 countries**	**Literacy (%) 2002**		**Enrolment (%) 2001/2002**	
		Female	Male	Female	Male								Male	femal	Male	female

		LEBf	LEBm	ELHf	ELHm	MMR	DASA	PWRP	CUW	IMR	PPP	HDI	Lm	Lf	Enm	Enf

Algeria	Alg	71,1	68	9,6	7,9	140	77	58	5	39	85	108	78	59,6	72	69
Egypt	Egy	70,8	66,6	8,8	7,4	84	46	53	11	35	218	120	67,2	43,6	80	72
Jordan	Jor	72,4	69,6	10,9	9	41	87	80	5	27	205	90	95,5	85,9	76	77
Lebanon	Leb	75	71,8	10,4	8,4	150	45	85	3	28	274	80	92,4	81	77	79
Morocco	Mor	70,3	66,6	11,9	9,4	220	40	45	9	39	49	125	63,3	38,3	61	52
Oman	Oma	74,3	70,9	11,1	8,3	87	92	98	24	11	137	74	82	65,4	62	63
Saudi Arabia	Sau	73,6	71	11	8,6	23	90	87	14	23	153	77	84,1	69,5	58	57
Syria	Syr	73	70,5	10,5	8,5	160	67	33	7	23	142	106	91	74,2	62	57
Tunisia	Tun	74,8	70,7	10,3	8,2	120	90	71	4	21	70	92	83,1	63,1	74	75

Medium Human Development (MHD)

Additional information is obtained by using the figure 1 vertically, and comparison can be made according to variables DASA, PWRP and Enm which have the highest relative contribution to the second axis (F2). For instance, in the first group (LHD), Djibouti, Mauritania and Yemen have comparable low human development but the percentage of deliveries attended by skilled personnel in Djibouti (79) is double that of Mauritania (40) and five times that of Yemen (16). A similar comparison can be made in the third group (MHD), between countries having 90% of deliveries attended by skilled personnel (Saudi Arabia, Oman) and those for which this percentage is less than 46% (Egypt, Morocco). Finally, in the second group (HHD), Libya with 76% of deliveries attended by skilled personnel is opposed to Bahrain, Kuwait, Qatar and United Arab Emirates achieving respectively 94%, 99%, 97% and 96%.

**Figure 4:**
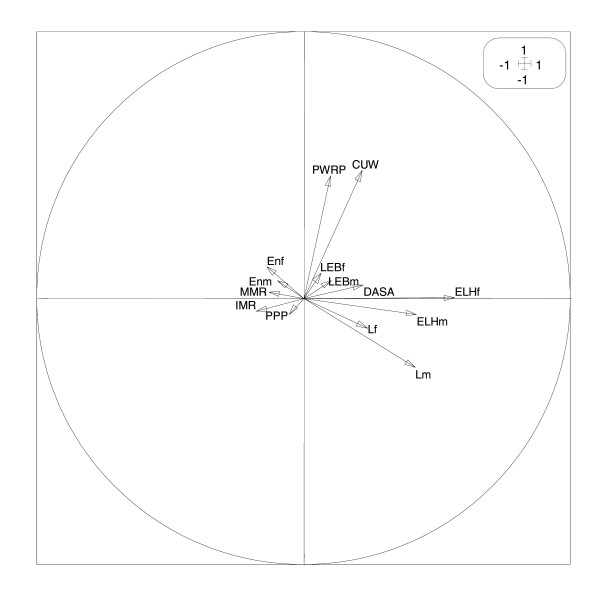
**Correlations on the second plane**. Correlation circle of indicators on the second factorial plane (F3 × F4) of the principal components analysis.

Taking into account the information extracted from the first plane, we may seek additional information from the third axis (F3) which can be seen as the axis of lost health since the main contribution to this axis is provided by ELHf and ELHm (see Figure 4). Here, independently of the level of global human development, we find Yemen with the maximum of lost healthy years, opposed to Egypt with the minimum.

**Figure 5:**
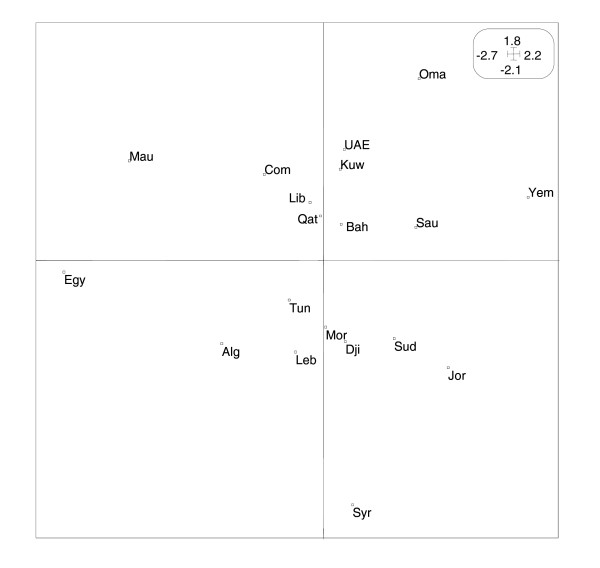
**Countries on the second plane**. Projection of countries on the second factorial plane (F3 × F4) of the principal components analysis.

Finally, children under weight (CUV) and pregnant women receiving prenatal care (PWRP) are the variables contributing the most to the fourth axis (F4). This axis provides a curious opposition between countries of the second group (HHD) with relatively high development index and high levels of children underweight (Oman (24), UAE (14), Kuwait (10)) opposed to countries of the third group (MHD), with very low levels of children underweight (Lebanon (3), Jordan (5), Algeria (5), Syria (7)) (see Figure 5).

## Discussion

Different authors have made comparisons between Arab countries and other regions. In particular, it was indicated that Arab countries lag behind the world's advanced and leading developing countries in terms of Science and Technology (S&T) [22,23]. In the present paper, we focus on the link between human development and health indicators. Beyond descriptive analysis showing disparities and similarities among Arab countries according to different indicators, our main purpose is to point out global and specific insufficiencies in order to propose pragmatic and efficient strategies that could improve human development in each country and in the Arab world as a whole region.

As indicated in the result's section, theoretically, the first group of countries (LHD) offers a multitude of opportunities of improvement in each component implied in the human development index (health, education, standard of living). In particular, this group which represents 20% of the Arab population has unacceptable levels of maternal and child mortality. Concretely, however, improvement of health indicators is conditioned by economic and political constraints (low income, military conflicts and drought).

On the opposite side, although the second group of rich countries (HHD) has still few possibilities of improvement, notably in terms of infant and maternal mortality, the results will have very limited impact of the whole region since this group represents only 3% of the Arab population. Moreover, the gain will be somehow offset by the burden of injuries and non communicable diseases which are exponentially increasing in the rich gulf countries [24-27].

A high potential of improvement is offered by the third group (MHD). Indeed, in this group representing 2/3 of the Arab population, human development level is lower than income level and many indicators can be improved. The quasi-totality of countries have high maternal and infant mortality levels contrasting with their economic development. Many countries have unacceptable low percentages of deliveries attended by skilled personnel and/or percentages of pregnant women receiving prenatal care. In particular, rural populations are generally deprived of access to health care and facilities.

Now, how can the Arab world as a whole region, by group or by country, secure the positive achievements and remedy to the shortages? Different publications have dealt with this issue. As indicated by the United Nations Population Fund [17], despite improvements to the health services made available to the populations of Arab countries and the ensuing reduction in mortality, the crude death rate, infant and child mortality rates and, consequently, the maternal mortality ratio, remain extremely high. According to this report, the main reasons behind the persistently high infant and maternal mortality levels are pregnancies at young age, pregnancies at a late age, and pregnancies that are too closely spaced.

Although Arab countries share many cultural practices, such as marriage at young age, multipart and consanguity, maternal mortality ratios and infant mortality rates still vary markedly between countries. For infant mortality, socio-demographic, perinatal and economic factors were considered in a study concerning 16 Arab countries [28]. Egypt, Morocco, Sudan, Yemen and Iraq were classified in the group with the highest infant mortality rates but attention was drawn to the impact of the demographic, social, perinatal care and economic factors on infant health in the whole region. Maternal and infant mortality are challenging decision makers of developing countries in general and those of the Arab world in particular [29,30]. As indicated earlier, in many countries, women are still assisted in delivery by traditional birth attendants or only by relatives and many of them deliver alone. Beyond the difficult accessibility to health centres especially for rural women, the problem seems more complex. According to the author of a study in Islamic and Arabic countries [31], the low social and economic status of girls and women is a fundamental determinant of maternal mortality in many Islamic and Arab countries. Whereas a study in the Middle East and North Africa (MENA) region indicated that haemorrhage, septicaemia and obstruction of the vaginal canal are the most frequent causes of childbirth-related death; and these risks could be reduced to a minimum through the prompt provision of skilled assistance [32]. In the same direction, an alarming publication was recently released by a group of Moroccan physicians under the supervision of seven medical associations [33]. According to these authors, the maternal mortality ratio in Morocco has not improved during the last seven years, decreasing only from 228 in 1997 to 227 in 2004, with 60% of mortality occurring in rural areas. Lack of antenatal and prenatal care, inaccessibility to health centres, absence of qualified staff and operational equipment were cited among the causes behind this unacceptable level of maternal mortality, which, by the way, is also correlated to a high level of infant mortality.

Other problems raised by different authors include: overworked and unqualified nurses and midwives offering ill-services and humiliating interactions, social inequalities and health inequity depriving the poor categories from access to health facilities and care [8], and finally, as indicated by the last report released by Transparency International (TI), corruption is affecting health public services in many Arab countries[34]. According to a TI-Morocco survey, four out of five people interviewed in 2002 described corruption in the public health system as 'common to very common'. More generally, the impact of corruption on human development in the Arab World was also considered [35].

## Conclusion

Arab countries have made substantial economic and social progress during the last decades. The post colonial era has known significant improvement in life expectancy and reduction in maternal and infant mortality. However, considering its natural wealth and human resources, the Arab world has accomplished less than expected in terms of human development. Huge social inequalities and health inequities exist inter and intra Arab countries.

The present paper was mainly devoted to the relationship between health indicators and human development in the Arab world. The three groups of countries obtained by the use of Principal Components Analysis agree fairly with the classification of the United Nations Development Programme. Although income (GDP) was not included in the analysis, the countries: Comoros, Djibouti, Mauritania, Sudan and Yemen were seen to have similar human development, sharing in particular, low levels of life expectancy and high infant and maternal mortality. On the opposite side, the oil-rich countries (Bahrain, Kuwait, Libya, Qatar, UAE) seem similar in that they share high human development expressed in terms of high level of life expectancy, high levels of deliveries attended by skilled personnel and pregnant women receiving prenatal care, resulting in low levels of infant and maternal mortality. The remaining countries (Algeria, Egypt, Jordan, Lebanon, Morocco, Oman, Saudi Arabia, Syria and Tunisia) were classified in a heterogeneous medium human development group.

Analysing the deficiencies in each group, our study lead us to conclude that a high potential of improvement is offered by the third group. Consequently, the discussion on health indicators concentrated on the causes of high maternal and infant mortality and the ways to reduce them. Significant improvement can also be obtained by reducing the unacceptable levels of infant and maternal mortality in the first group. Making health services accessible to the least disadvantaged population especially in rural areas, tackling the absence of qualified staff and operational equipment, dealing with the low social and economic status of girls and women and investing in preventive health through early diagnosis, sensitisation and care will inevitably improve health conditions and enhance human development in the Arab region and for the general population.

## Competing interests

The author(s) declare that they have no competing interest.

## Authors' contributions

The two authors contributed equally.

## Dedication

This paper is dedicated to the Arab children deprived of health care, in particular those living in Palestine and Iraq.
